# Description of *Callistethus hamus* sp. nov. (Coleoptera, Scarabaeidae, Rutelinae) from continental Southeast Asia using synchrotron to illustrate the aedeagus

**DOI:** 10.3897/zookeys.881.34821

**Published:** 2019-10-17

**Authors:** Yuan-Yuan Lu, Carsten Zorn, David Král, Ming Bai

**Affiliations:** 1 Key Laboratory of Zoological Systematics and Evolution, Institute of Zoology, Chinese Academy of Sciences, 1 Beichen West Road, Chaoyang District, Beijing 100101, China Institute of Zoology, Chinese Academy of Sciences Beijing China; 2 Rostocker Strasse 1a, Gnoien 17179, Germany Unaffiliated Gnoien Germany; 3 Department of Zoology, Faculty of Science, Charles University, Viničná 7, CZ-128 43, Praha 2, Czech Republic Charles University Praha Czech Republic

**Keywords:** Anomalini, *
Callistethus
*, China, Laos, Vietnam, new species, Rutelinae, 3D models

## Abstract

A new species, *Callistethus hamus* Lu & Zorn, **sp. nov.**, is described from China, Laos, and Vietnam. Additionally, we used synchrotron (Shanghai Synchrotron Radiation Facility) to scan the aedeagus. The virtual 3D model of the aedeagus is reconstructed and provided.

## Introduction

The genus *Callistethus* Blanchard, 1851, includes, to date, more than 150 species (Krajčík 2012; Filippini 2016). Only six species and subspecies were previously recorded from China: *Callistethus auronitens* (Hope, 1835), *C. excisipennis* Lin, 1981, *C. formosanus* Kobayashi, 1987, *C. plagiicollis plagiicollis* (Fairmaire, 1886), *C. p. isidai* Miyake, 1987, and *C. stoliczkae* (Sharp, 1878). When working on anomaline specimens from the IZAS collection, we found one species from Yunnan which appeared to be *Callistethus rachelae* (Arrow, 1917), originally described from Myanmar. However, after examination of the type material of this species, deposited in the NHMUK, it became apparent that there are two different species; the Yunnan species proved new to science. Additional specimens of the new species were later found also from Laos and Vietnam and the species is described herein.

Recently, Micro-CT and synchrotron radiation microtomography techniques have been used in several extant and extinct insect groups, e.g., Formicidae of Hymenoptera (Staab et al. 2018), †Alienoptera (Bai et al. 2018, 2016), Geotrupidae of Coleoptera (Bai et al. 2017), and Platycnemididae of Odonata (Steinhoff and Uhl 2015). These techniques enable a new method of acquiring the internal and external 3D morphology. In Rutelinae, the aedeagus plays a very important role in the species identification. This organ has usually a strongly three-dimensional shape in this subfamily. Therefore, sometimes it is difficult to demonstrate the precise morphology of the aedeagus using traditional line drawings or 2D photographs, although several planes could give an approximate idea of the spatial structure. Here we use synchrotron technique to provide 3D model of the aedeagus in this new species, which can provide more morphological information for the future studies of Rutelinae.

## Materials and methods

The morphological terminology follows Lu et al. (2018).

The body length was measured from the apex of the clypeus to the apex of the elytra. The length of pronotum was measured in the middle in dorsal view, its width at the greatest width. The ratio of interocular width to head width was measured in dorsal view at greatest width of head and nearest interocular distance.

For observation of morphological structures, some specimens were softened by soaking the aedeagus in detergent for ca. 24 hours. Observations and dissections were carried out under an Olympus SZ61 stereomicroscope and a Zeiss Stemi 2000. The digital images were taken with a Canon 5D digital camera in conjunction with a Canon MP-E 65 mm f/2.8 1–5× Macro Lens, and then stacked by Helicon Focus 5.3.10. All images were edited and adjusted in Adobe Photoshop CS6 Extended. The distribution map was created by QGIS 3.4 software (QGIS Development Team). Coordinates and altitude were assigned for each locality mentioned in the text (material examined in each species). These data were used in the construction of distribution maps (see Fig. 24).

The aedeagus of one specimen was imaged using propagation phase-contrast synchrotron radiation microtomography (PPC-SR-μCT) on the beamline 13W at the Shanghai Synchrotron Radiation Facility (SSRF). The isotropic voxel size was 3.25 μm. The beam was monochromatised at an energy of 14 keV using a double crystal monochromator. To obtain a phase-contrast effect, we used a sample-detector distance (propagation distance) of 300 mm and 900 projections on 180°. The phase retrieval and slice reconstruction were performed using PITRE software. Based on the obtained image stacks, three-dimensional structures of the specimen were reconstructed and virtually dissected with Amira 5.4 (Visage Imaging, San Diego, USA) (see Figs 19–23 and Appendix 1). All images were edited and adjusted in Adobe Photoshop CS6 Extended.

Type specimens of the new species are provided with one red printed label “*Callistethus hamus* sp. nov., HOLOTYPE [or] PARATYPE, Lu & Zorn, 2019”.

The material examined is housed in the following collections (curators in parenthesis):

**CZPC** Carsten Zorn private collection, Gnoien, Germany

**FWPC** Falei Wang private collection, Chongqing, China

**IZAS** Institute of Zoology, Chinese Academy of Sciences, Beijing, China (Ming Bai)

**MSPC** Matthias Seidel private collection, Prague, Czech Republic

**NHMUK** Natural History Museum, London, United Kingdom (Maxwell W. L. Barclay, Michael Geiser)

**NMEC** Naturkundemuseum Erfurt, Germany (Matthias Hartmann)

## Taxonomy

### Callistethus hamus

Taxon classificationAnimaliaColeopteraRutelidae

Lu & Zorn sp. nov.

19BBF8B0-7B54-5397-9247-DFD82C748143

http://zoobank.org/03158F0F-19B4-4940-8025-649989535595

Figs 1–9 , 19–23

#### Type locality.

China, Southern Yunnan, 23 km NW of Jinghong, vicinity of Nan Ban, Xishuangbanna [Prefecture], 22°09.49"N, 100°39.92"E, 730 m.

#### Material examined.

**Holotype (HT).** CHINA • ♂; Yunnan Province, Dai Autonomous Prefecture of Xishuangbanna, Jinghong City, Na Ban River Watershed National Nature Reserve; 22°09.49'N, 100°39.92'E; 20 May 2008; A Weigel leg.; NMEC.

#### Paratypes.

CHINA • 1 ♀; same data as the holotype • 1 ♂; Yunnan Province, Xishuangbanna, Jinghong, Na Ban River Watershed National Nature Reserve; 22°09.49'N, 100°39.92'E; 5 Jun. 2008; A. Weigel leg.; NMEC; [Micro-CT specimen] • 1 ♂ 1 ♀; Yunnan Province, Xishuangbanna, Jinghong, Na Ban River Watershed National Nature Reserve; 22°09.49'N, 100°39.92'E; 5 Jun. 2008; A. Weigel leg.; CZPC • 1 ♀; Yunnan Province, Xishuangbanna, Jinghong, Na Ban River Watershed National Nature Reserve; 22°09.49'N, 100°39.92'E; 15 Jun. 2008; NMEC • 1 ♀; Yunnan Province, Xishuangbanna, Jinghong, Na Ban River Watershed National Nature Reserve; 22°09.49'N, 100°39.92'E; 5 Jun. 2008; A. Weigel leg.; NMEC • 1 ♂; Yunnan Province, Xishuangbanna, Jinghong, Na Ban River Watershed National Nature Reserve; 22°09.49'N, 100°39.92'E; 12 May 2008; A. Weigel leg.; NMEC • 1 ♀; Yunnan Province, Xishuangbanna, Jinghong, Na Ban River Watershed National Nature Reserve; 22°09.49'N, 100°39.92'E; 16 May 2009; Malaise trap; L.Z. Meng leg.; IZAS IOZ(E)1966878 • 1 ♀; Yunnan Province, Xishuangbanna, Jinghong, Na Ban River Watershed National Nature Reserve; 22°07.85'N, 100°40.12'E; 26 May 2009; Malaise trap; L.Z. Meng leg.; IZAS IOZ(E)1966879 • 1 ♀; Yunnan Province, Xishuangbanna, Jinghong, Na Ban River Watershed National Nature Reserve; 22°09.49'N, 100°39.92'E; 16 May 2009; flight interception traps; L.Z. Meng leg.; IZAS IOZ(E)1966880 • 1 ♀; Yunnan Province, Xishuangbanna, Jinghong, Na Ban River Watershed National Nature Reserve; 31. Jul. 2013; FWPC • 1 ♂; Yunnan Province, Menghai, Menghun Town; 21°50.48'N, 100°23.15'E; 4 Jun. 1958; L.Y. Zheng leg.; IZAS IOZ(E)1966104 • 1 ♀; Yunnan Province, Xishuangbanna, Menghai, Menghun Town; 21°50.48'N, 100°23.15'E; 1 Jun. 1958; X.W. Meng leg.; IZAS IOZ(E)1966106 • 1 ♀; Yunnan Province, Xishuangbanna, Menghai, Menghun Town; 21°50.48'N, 100°23.15'E; 4 Jun. 1958; L.Y. Zheng leg.; IZAS IOZ(E)1966107 • 1 ♀; Yunnan Province, Xishuangbanna, Menghai, Mengzhe Town; 21°59.15'N, 100°16.02'E; 8 Jul. 1958; S.Y. Wang leg.; IZAS IOZ(E)1966105.

LAOS • 1 ♂; Vientiane Province, Phou Khao Khoay; 18°24.15'N, 103°02.15'E; 4–17 May 2005; P. Moravec leg.; CZPC.

VIETNAM • 4 ♂♂ 2 ♀♀; Lâm Đồng Prov., Dambri, Bảo Lâm; 11°38.42'N, 107°44.52'E; May 2017; local collector leg.; CZPC • 1 ♀; Lâm Đồng Prov., Bảo Lộc; 11°32.88'N, 107°48.46'E; March 2018; local collector leg.; CZPC • 5 ♂♂ 1 ♀♀; Lâm Đồng Prov., Bảo Lộc; 11°32.88'N, 107°48.46'E; March 2018; local collector leg.; CZPC • 1♂ 1 ♀; Lâm Đồng Prov., Bảo Lộc; 11°32.88'N, 107°48.46'E; March 2017; local collector leg.; MSPC.

**Description of holotype (♂).** Body shape elongate ovoid, convex.

***Colour*.** Head including antenna orange-brown, with two small black spots on vertex; pronotum orange-brown with two moderately large black spots in the middle of each side; an additional longitudinal stripe near the lateral margin (not reaching anterior and posterior margins) separating the yellow sides from the orange disc (Figs 1, 4); elytra light reddish-brown with black markings as follows: an elongate spot on humeral callus, another smaller spot beneath shoulder at lateral margin, a zig-zag band of loosely connected spots crossing the middle of elytra, another zig-zag band shortly before the posterior margin (Fig. 1); propygidium black with a transverse yellow spot at the outer posterior margin (Fig. 5); pygidium black with a narrow yellow longitudinal middle line (Fig. 5); underside yellow with black markings, with slight metallic shine (Fig. 2); black macula in the middle and at extremities of femurs; various black markings present on all tibiae; pro- and mesotarsi brown, metatarsus black; major part of meso- and metaventrite black; meso-metaventral process yellow; major part of abdominal ventrites 1-3 yellow, abdominal ventrites 4-6 black with lateral yellow spot (Fig. 2).

**Figures 1–9.**
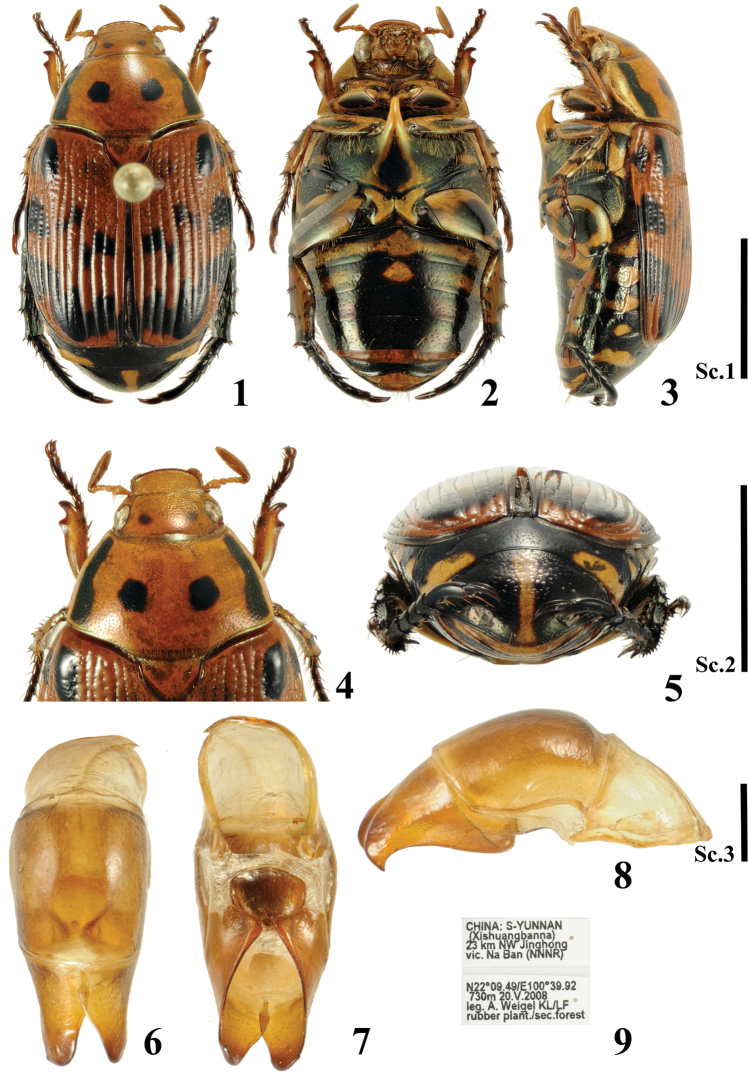
Holotype of *Callistethus hamus* Lu & Zorn, sp. nov. **1–3** habitus **1** dorsal view **2** ventral view **3** lateral view from left **4** head and pronotum **5** propygidium and pygidium **6–8** aedeagus **6** dorsal view **7** lateral view from right **8** ventral view **9** label. Key: Sc. 1, Scale bars: 5 mm (**1–3**); Sc. 2, 5 mm (**4, 5**); Sc. 3, 1 mm (**6–8**).

***Head.*** Clypeus subtrapezoidal, anterior angles rounded; anterior margin weakly reflexed, very densely, transversely rugo-punctate; frons rugo-punctate, with very shallow impression in the middle, confluently punctate at sides; vertex finely and sparsely punctate in the middle, more coarsely punctate laterally; ratio interocular width/width of head approximately 0.71; antennal club longer than antennomeres 1–6 combined (Figs 1, 4).

***Pronotum*** approximately 1.89 times wider than long; sparsely and finely punctate, punctures very fine on disc, becoming gradually larger toward the sides; surface with additional micropunctures; anterior angles sub-rectangular; posterior angles obtuse; broadest at base; sides evenly curved in the middle, straight and strongly converging before anterior angles; sides very slightly sinuate near posterior angles; basal marginal line only present near posterior angles; anterior marginal line indistinct in the middle (Fig. 4).

***Scutellum*** subtriangular, slightly broader than long, finely and sparsely punctate (Fig. 4).

***Elytra*** regularly striate; primary striae and secondary stria of subsutural interstice sulcate in posterior half, therefore intervals flat in anterior half and gradually more convex posteriad; strial punctation distinct, coarse; 2^nd^ and 3^rd^ interstices each with an indistinct row of punctures; elytral surface with additional sparse micropunctation; humeral umbone and apical protuberance rather prominent; epipleuron broad near humerus, ending approximately at the middle of elytron; posterior margins evenly, separately rounded; apico-sutural angle forming small dent (Fig. 1).

***Pygidium*** convex; apex broadly rounded; moderately densely, coarsely punctate; apex with several long, erect brownish setae (Fig. 5).

***Ventral thoracic surface*** smooth (Fig. 2).

***Meso-metaventral process*** very long, reaching middle of procoxae; projecting upward in lateral view; apex acute (Fig. 3).

***Abdominal ventrites*** with transverse row of rather sparse brown setae (usually only in lateral part); ventrites 1 and 2 carinate laterally (Figs 2, 3).

***Legs.*** Mesofemur with two bands of long brown setae: one along anterior margin; another transverse row of punctures parallel to posterior margin. Protibia bidentate, rather slender; proximal tooth short, situated close to the rather short, weakly outwards curved apical tooth; inner spur short, articulated in opposite to proximal tooth. Metatibia strongly fusiform; protarsomere 5 shorter than tarsomeres 1–4 combined in all legs; inner protarsal claw slightly widened and deeply incised apically, lower margin with obtuse angle basally, upper branch spiniform; outer mesotarsal claw long, curved, deeply incised at apex, upper branch spiniform; metatarsal claws somewhat unequal, outer claw distinctly broader and longer than inner (Figs 1–3).

***Aedeagus.*** Parameres short, with the hook-like apex in lateral view. See Figs 6–8.

**Female.** Abdominal ventrites 4–6 with extensive yellow markings. Apical tooth of protibia long and somewhat spatulate; protarsus articulated slightly basally of level of proximal tooth; inner spur long; protarsomere 1 as long as combined length of protarsomeres 2–4; antennal club short, only slightly longer than antennomeres 2–6 combined.

#### Measurements.

Total body length 11.5–14.4 mm (HT 11.8 mm), total body width 6.5–8.4 mm (HT 7.5 mm).

#### Morphological variability.

Vertex with or without two black spots. Elytral spots vary slightly in shape and size. The extend of black markings of the ventral side variable. The secondary longitudinal rows of punctures in interstices 2 and 3 sometimes distinct until the posterior half. Shape of parameres very consistent (Figs 19–23, Appendix 1).

#### Differential diagnosis.

**Figures 10–18.**
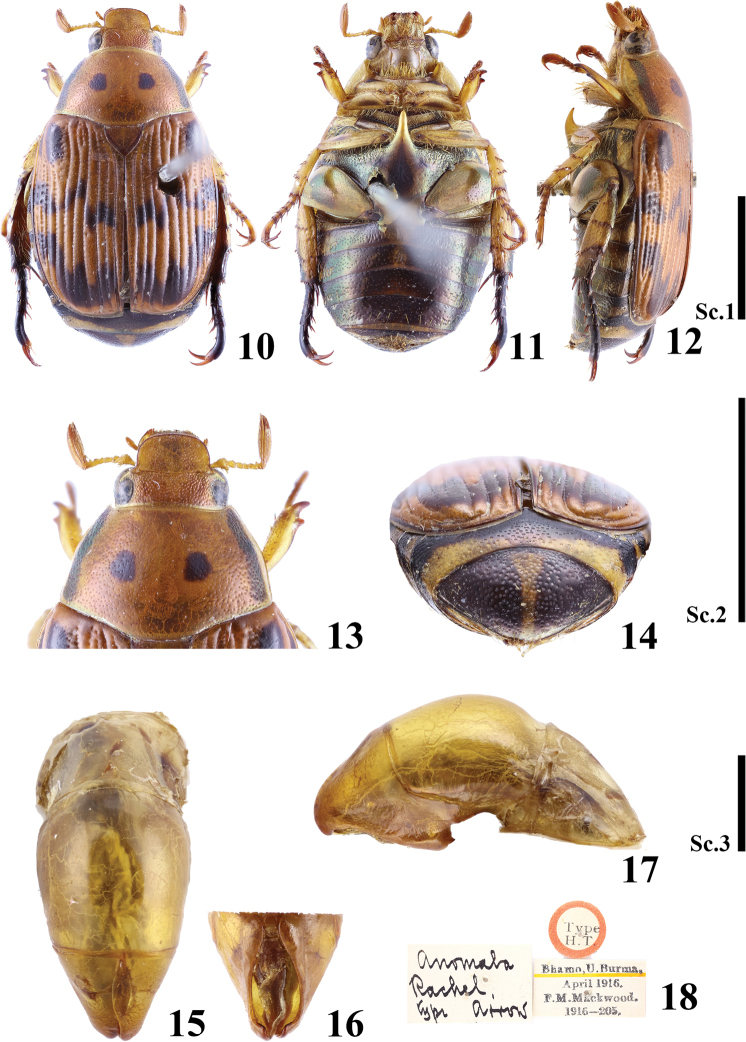
Holotype of *Callistethus rachelae* (Arrow, 1917). **10–12** habitus **10** dorsal view **11** ventral view **12** lateral view from left **13** head and pronotum **14** propygidium and pygidium **15–17** aedeagus **15** dorsal view **16** lateral view from right **17** ventral view **18** label. Key: Sc. 1. Scale bars: 5 mm (**10–12**); Sc. 2, 5 mm (**13, 14**); Sc. 3, 1 mm (**15–17**).

*Callistethus hamus* sp. nov. resembles several other, similarly coloured South East Asian species of this genus. The reddish colour combined with black markings is also found in *C. maculatus* (Guérin-Méneville, 1834), *C. picturatus* (Candèze, 1869), *C. rachelae* (Arrow, 1917), *C. spiniferus* (Ohaus, 1915), and *C. stolidopygus* (Ohaus, 1915). The new species is most similar to *C. rachelae*, which also has only two black spots on the pronotum, not four as all other red species (Figs 10–18). *Callistethus hamus* sp. nov. differs from *C. rachelae* primarily in the shape of the aedeagus (Figs 6–8, 15–17). The ventral margin of the parameres of *C. rachelae* is somewhat membranous and soft, but fully sclerotised in *C. hamus* sp. nov. The apices of the parameres in *C. hamus* sp. nov. are longer and more strongly curved compared to those of *C. rachelae* (Figs 15–17).

**Figures 19–23.**
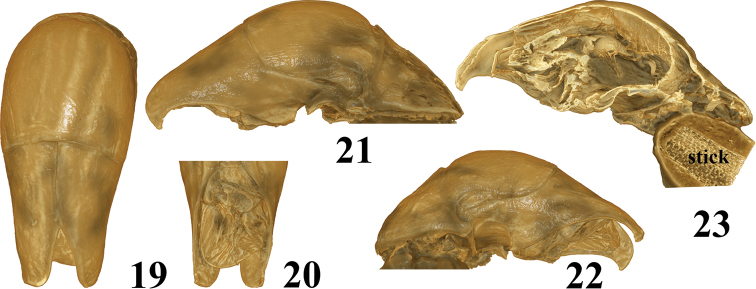
3D model for aedeagus of *Callistethus hamus* Lu & Zorn, sp. nov. Paratype from China, Yunnan. These photos are screen shots of the 3D model of the aedeagus.

#### Etymology.

The specific epithet refers to the hook-like shape of the parameres of the new species.

#### Collecting data.

Specimens collected in the Naban River Watershed National Nature Reserve by Lingzeng Meng and Andreas Weigel were collected with cross-window traps in the tree canopy and malaise trap.

#### Distribution.

**Figure 24.**
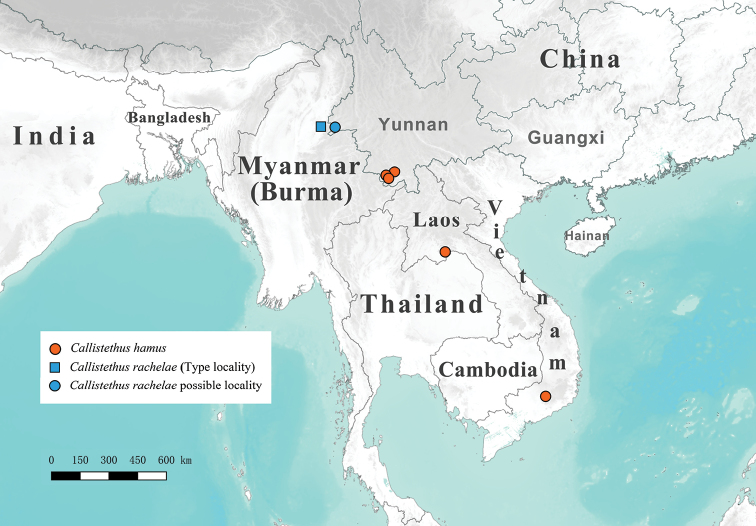
Map of Southeast Asia showing distributions of *Callistethus hamus* and *Callistethus rachelae*.

China (Yunnan), Laos (Vientiane), Vietnam (Lâm Đồng) (Fig. 24).

## Discussion

We found one female specimen in the collection of the IZAS, collected at Jinghan Town, Dehong prefecture, Yunnan, which could represent the first record of *C. rachelae* in China. The collecting locality is very close to the type locality of *Callistethus rachelae* in neighbouring Myanmar (“Bhamo”, see Fig. 24). This specimen differs slightly from all specimens of *C. hamus* sp. nov. in having a slightly more slender meso-metasternal process, which is consistent with the holotype of *C. rachelae* sp. nov. However, male specimens are needed to confirm the presence of *C. rachelae* sp. nov. in China.

In the present study the synchrotron technology was used for the first time to study a Rutelinae species. Normally, the original data of 3D information need to be present in some specific software (like Amira and Maya), which require a higher hardware configuration. Obviously it will limit the widespread use of this technology by taxonomists to some extent. In this study, we found that the structure complexity of the aedeagus of the here examined Rutelinae is relatively low and can be obtained as 3D model in pdf format containing about 20Mb (Appendix 1). It can be easily opened in Adobe Reader Pro by enabling the playing 3D content checkbox. It is possible to zoom and rotate the 3D model as desired, which can enhance details. By comparing figures 6–8 with 19–21, we are sure that this 3D model reflects the real characters. Moreover, it can improve the identification accuracy compared to 2D images by adjusting the viewing angle.

Unfortunately, in this study, internal structures like the endophallus are not clearly visible in the 3D model. The synchrotron and Micro-CT technology require a dried out specimen and are suitable for sclerotised structures, while membranous structures like the endophallus will be out of shape during the operating steps. Therefore, it will be difficult to obtain the 3D structure of the endophallus. The disadvantages of this technology include also its high cost and the fact that the procedure is time-consuming.

In summary, the synchrotron and Micro-CT technology have great potential for wide use in the taxonomy of the Rutelinae and other insect groups, because it provides accurate morphological information of 3D structures. But the shortcomings of this technology are still obvious. Therefore, the innovation of new techniques that aid in the visualisation of microscopic anatomical structures is needed. The recently invented LED-SIM (DMD-based LED-illumination structured illumination microscopy) facilitates the acquisition of nano- and micro-3D structures of small organisms in a high-resolution format (500 nm in the XY-plane and 930 nm along the Z-axis) (Ruan et al. 2016). Recently, the second generation of LED-SIM could provide large-scale 3D imaging of insects in natural colour (Qian et al. 2019). Therefore, it is possible that the 3D imaging will be widespread in future.

## Supplementary Material

XML Treatment for Callistethus hamus

## Figures and Tables

**Figure 1A.**
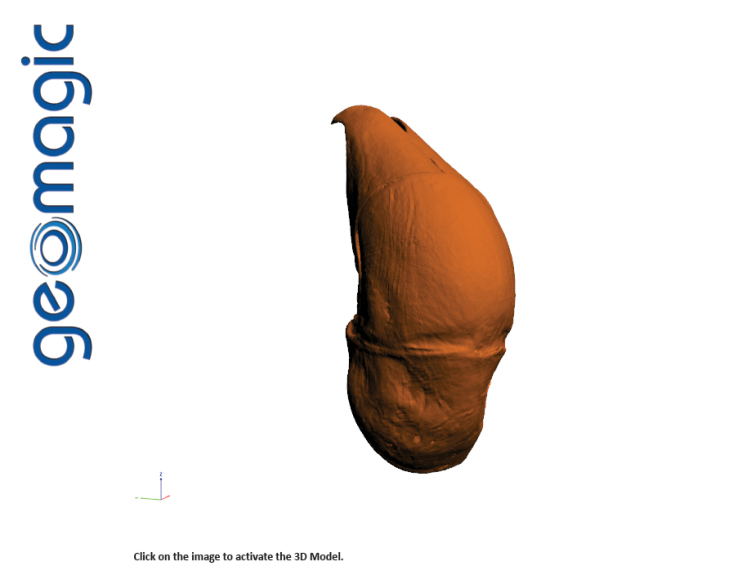
3D model for aedeagus of *Callistethus hamus* Lu & Zorn, sp. nov. in pdf format.
